# Psychological Distress in Alzheimer’s Disease Family Caregivers: Gender Differences and the Moderated Mediation of Resilience

**DOI:** 10.3390/healthcare11233084

**Published:** 2023-12-01

**Authors:** José Manuel Ponsoda, María Ángeles Beleña, Amelia Díaz

**Affiliations:** 1 Faculty of Education, University of Alicante, 03009 Alicante, Spain; josemanuel.ponsoda@ua.es; 2 Faculty of Psychology, University of Valencia, 46010 Valencia, Spain; mangeles.belena@uv.es

**Keywords:** gender, psychological distress, resilience, social support, Alzheimer disease family caregivers, burden

## Abstract

Different studies have reported gender differences in the variables of psychological distress, burden, social support, and resilience in family caregivers of relatives with Alzheimer’s disease; however, there is no clear evidence of the specific role of burden, social support, and resilience between gender and psychological distress. The aim of the present study is to clarify the role of these variables. Burden, psychological distress, social support, and resilience have been assessed in a cross-sectional design with a convenience sample of 140 family caregivers of relatives with Alzheimer’s disease. Our results confirm gender differences in terms of psychological distress, with higher scores found in female than in male caregivers, whilst male caregivers showed higher scores in terms of resilience than female caregivers. No gender differences have been found in terms of burden and social support. In addition, moderated mediation was obtained via the interaction of gender and social support on resilience, which plays a mediating role in psychological distress. Social support and resilience have shown a protective role concerning the mental health of caregivers, but female caregivers need higher social support to present a similar level of resilience to male caregivers.

## 1. Introduction

Recent decades have witnessed the increased ageing of the global population with a subsequent increase in age-associated diseases. It is estimated that between 2015 and 2050, the number of people over 60 years of age will more than double, with this subset of the population going from 900 million to 2 billion [1]. The WHO (World Health Organization) reported that 55 million people have dementia in 2023, with 10 million new cases being diagnosed every year [2]. If this prevalence remains constant, it is estimated that there will be around 150 million people affected by this pathology in 2050, becoming a serious public health problem [3].

Alzheimer’s disease (AD) is the more common type of dementia, accounting for 60–80% of people with dementia. It affects brain areas that deal with memory, attention, language, praxis, and executive functions, causing dramatic personality and behaviour changes [4]. Most people with AD are cared for by their relatives at home. Family members are the main source of help for those people who cannot take care of themselves, and it is mainly women who assume the role of primary caregivers in Spain [5].

Dementia presents a clear gender bias, both directly and indirectly. Directly because of the longer life expectancy of women and the higher incidence of dementia in old age; more women present with this disease; and indirectly, because women are the main caregivers of people with dementia, contributing around 70% of the care hours dedicated to people with dementia [2]. A systematic review of the profile of Alzheimer’s family caregivers over the last ten years in Spain performed in 2021 found that most caregivers were women (80.25%), between the ages of 50 and 60 years old, either the daughter or son of the relative with Alzheimer’s (52%), and most of them had an education level corresponding to secondary studies [6]. Additionally, caregiving seems to affect men and women differently, with greater negative impacts on women than men. There is a broad agreement that female caregivers present more psychological problems affecting their mental health and experience greater attrition than male caregivers [7,8]. Specifically, variables such as burden, psychological distress, anxiety, and depressive symptoms evaluated in caregivers of AD relatives show higher rates than their counterparts caring for people with other illnesses, but, in all cases, they are higher in women than in men [9,10,11,12,13].

Several approaches have been advanced to explain these gender differences in caregiving. Gender roles explanations highlight the traditional role of women as caregivers and men as income providers, where men are not assumed to become caregivers; however, when caregiving, due to the role of socialization, it is less probable that men will express or report difficulties or emotions associated with care activities [14,15]. A second approach bases their assumptions on the stress-coping theory, proposing that women, in relation to men, are more exposed to stressors, their appraisal of the caring situation is more negative, they use coping strategies inefficiently and less adaptatively, such as through denial, escape, or avoidance, and social support is less available or sought [15,16,17,18]. With respect to social support, the first and the second approaches can be combined in the case of women since women receive or seek less social support than men due to the restrictions associated with their caregiving role [9,19]. A third theory focuses on the personality factor of neuroticism, which is higher in women than men, as an explanation of why women as caregivers present higher levels of depression, anxiety, and other psychiatric symptoms than men [20]. Finally, gender differences could be explained by other mediating variables such as age, marital status, socioeconomic level, education, culture, or aspects related to the care recipient, such as relationship or the stage of AD [21]. However, although most of the studies have confirmed gender differences in caregiving, there is a small group of studies that did not find gender differences [22,23].

Researching protective variables that decrease the impact of caring for a relative with AD on the mental health of caregivers led to the study of mediating or moderating variables. Resilience, optimism, social support, and coping strategies are proposed as protective elements that could reduce the most frequent negative consequences of caregiving: burden, anxiety, and depression [24,25,26,27,28,29]. Both the subjective or perceived burden and the objective burden, measured as daily hours dedicated to care, are among the variables more clearly associated with a higher risk of mental health problems, which are normally higher in female caregivers [30]. Additionally, in the specific case of family caregivers of AD, social support, as a source of emotional, instrumental, and informative support, has been shown to have mediating effects on psychological distress in different studies, with both female and male caregivers [30,31], or with female caregivers only [32]. Resilience, as a positive adaptation to face adversity, has also been studied in caregivers as a protective variable [33,34,35]. Resilience and social support, together or alone, have been found to present a mediating or moderating role between burden and psychological distress, usually controlling for sociodemographic variables [24,25,26,27,28,29]. Surprisingly, although female caregivers present higher social support and resilience than male caregivers, even considering the protective effects of these variables, the negative impact of caregiving on the mental health of women is higher than in men [32]. However, no study seems to have been performed taking gender as a relevant and important independent variable. Therefore, the basic questions in this context are as follows: first, why are female caregivers showing higher social support and resilience than male caregivers presenting with worse mental health? Additionally, more specifically, what is the effect of social support and resilience between gender and psychological stress?

Based on the above, the objective of this work was to study the effect of gender on the variables of burden, psychological distress, social support, and resilience. Specifically, the aim was to study the effect of gender in the mediating or moderating effect of burden, social support, and resilience between gender and psychological distress in the caregivers of relatives with AD.

## 2. Materials and Methods

### 2.1. Participants

The participants were 140 family caregivers of people with AD in the three provinces of the Valencian Community, a region in the east of Spain. Regarding gender, 96 were female and 44 were male caregivers. The age range was 18–91 years old. The inclusion criteria were as follows: (1) the relative receiving care has been diagnosed with AD, and (2) the relative with dementia is living in the community.

### 2.2. Measures

#### 2.2.1. Psychological Distress

Psychological distress was assessed with the 12-item General Health Questionnaire, GHQ-12 [36]. In this study, the items were scored using the Likert method that assigns a weight to each score (0-1-2-3) and the Standard GHQ score method (0-0-1-1), with the highest score indicating the greatest level of distress. The Likert score was used in all the analyses, except for the calculation of the percentage of people with and without distress, where the Standard GHQ score was used. As reported by Lundin et al. [37], the best threshold to discriminate cases of distress from those that are not for the GHQ Inx was ≥4 (sensitivity = 81.7 and specificity = 85.4). In this study, Cronbach’s α of 12 items scored based on the Likert method was 0.87, and for the Standard GHQ method, it was 0.87.

#### 2.2.2. Burden

The Zarit Burden Interview [38] method, ZBI, was used to assess caregiver burden. For each of the 22 items, respondents reported their perceived strain associated with the provision of care on a Likert scale ranging between zero (never) and four (nearly always). We computed the total scores and applied standard cut-offs [28] of low (≤20), mild to moderate (21–40), moderate to severe (41–60) and severe burden (≥61) in frequency analysis. Cronbach’s α was 0.85 in this study.

#### 2.2.3. Perceived Social Support

DUKE.UNC, Functional Social Support Questionnaire [39] includes 11 items and measures perceived social support. The item response options are on a 5-point scale ranging from 1 (much less than I would like) to 5 (as much as I would like). Higher scores indicate greater perceived social support. Cronbach’s α was 0.89 in this study.

#### 2.2.4. Resilience

The 25-item Connor–Davidson Resilience Scale (CD-RISC) [40] is a self-rated instrument to measure resilience as an ability to thrive in the face of adversity. This 5-point response scale ranges from 0 to 4, with higher scores indicating greater resilience. Cronbach’s α was 0.85 in this study.

### 2.3. Design and Procedure

The design is cross-sectional and uses a convenient sample. The participants came from Alzheimer Family Caregivers Association Centres. All of them signed the informed consent form and individually they completed the scales on a voluntary basis. The questionnaires and datasheet of each participant were assigned a number in order to preserve his/her anonymity. The permission to conduct this research was obtained from both the Alzheimer Association Centres and the Ethical Committee for Scientific Research of the University of Valencia (H1367489852167).

### 2.4. Statistical Analysis

IBM SPSS, version 23.0 (IBM Corporation, Armonk, NY, USA) software and PROCESS macro [41] for SPSS 23 were used for data analysis. Chi-squared, ANOVA, Student’s *t*, Cohen’s *d*, correlations, and mediation and moderation analyses were used in this study. Chi-squared analyses determined the possible significant differences between the percentages of female and male caregivers experiencing low and high psychological distress and mild/moderate/severe burden. ANOVA analysis showed the gender differences in terms of psychological distress, burden, social support and resilience. Finally, mediation and moderation analyses were performed to determine the mediated effect of resilience and the mediator effect of gender and social support in terms of psychological distress.

## 3. Results

### 3.1. Demographic Data

Table 1 shows information relating to the demographic variables. The age difference between the male and female caregivers was not statistically significant: men: Mean = 58.81; SD = 13.90; women: mean = 54.55; SD = 10.95); *t* (138) = 1.80; *p* = 0.07. About education, almost half of the sample had a primary level and the remaining had secondary and university education levels. Additionally, most caregivers were married or living with a partner. Finally, the family relations with the AD relative were daughter/son (67.9%), wife/husband (17.1%), daughter/son in law (9.3%), granddaughter/son (3.6%) and niece/nephew (2.1%). According to the three stages of progressing Alzheimer’s disease proposed by Feldman and Woodward [42], almost half of the care recipients were in stage II, and the other half were in stages I and III. The AD diagnostic and specific AD stage classification were performed by the health centres neuropsychiatrist.

### 3.2. Differences in the Variables

The prevalence of mental distress, when scoring using the GHQ12 ≥ 4, was 50.7%; 56.2% for female caregivers and 38.6% for male caregivers, which is a statistically significant difference in percentages, χ^2^ (1, *N* = 140) = 3.80, *p* = 0.05. In a similar way, concerning burden, when using the standard cut-offs of low (≤20), mild to moderate (21–40), moderate to severe (41–60) and severe burden (≥61), no caregiver presented low burden, 32 caregivers (22.9%) showed mild to moderate burden, 76 (54.3%) presented moderate-to-severe burden and, finally, 32 (22.9%) showed severe burden. There were not significant differences between female and male caregivers in burden χ^2^ (2, *N* = 140) = 1.95, *p* = 0.378.

The ANOVA performed using gender as the factor and psychological distress, social support, and resilience as dependent variables showed significant gender differences in terms of psychological distress (*F*(1.139) = 4.52; *p* = 0.035; partial *ɳ*^2^ = 0.030) and resilience (*F*(1.139) = 4.85; *p* = 0.029; partial *ɳ*^2^ = 0.034) but were not statistically significant for social support (*F*(1.139) = 0.29; *p* = 0.590) or burden (*F*(1.139) = 0.69; *p* = 0.409).

Table 2 presents, in more detail, the means, standard deviations, Student’s *t*, and Cohen’s *d* between male and female caregivers concerning the variables of psychological distress, social support and resilience. Male caregivers showed significantly higher resilience and lower psychological distress than female caregivers, with a difference close to half that of the standard deviation. Similar to the ANOVA analysis showed before, social support and burden did not present significant differences between male and female caregivers.

The differences in psychological distress, burden, social support, and resilience between caregivers looking after a relative at different stages of AD were analysed using Bonferroni post hoc test, but no significant differences were found in our sample.

### 3.3. Relationships between the Variables

Looking at the relationships between psychological distress, burden, social support, and resilience presented in Table 3, a clear pattern appears in the female sample. On one side, social support and resilience and, on the other side, burden, and psychological distress, correlating positively and significantly between them, but negatively when concerning the first and the second group of variables. In the case of male caregivers, only three relationships are significant, the positive relation between burden and psychological distress, and the negative ones between burden and social support and between psychological distress and resilience.

**Figure 1:**
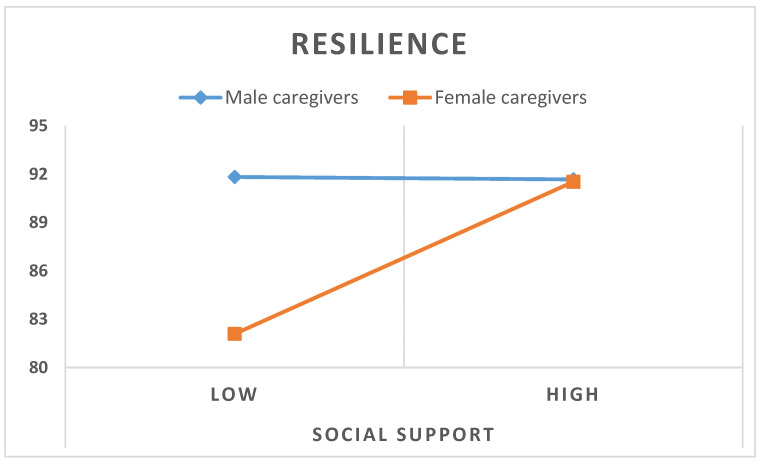
Interaction of social support group (high and low) and resilience scores.

Due to the lack of relationship between social support and resilience in male caregivers, in addition to the high and significant relationships of these variables in female caregivers, and in order to find possible moderation variables, ANOVA analysis was performed using gender (male/female) and social support (high/low, using the median as a cut-off point) as factors and resilience as the dependent variable. The result showed that the main effect of gender was statistically significant, *F*(1,1366) = 6.35, *p* = 0.013, and partial *η*^2^ = 0.045. The main effect of social support was statistically significant, *F*(1,136) = 5.61, *p* = 0.019, partial *η*^2^ = 0.040, also. Finally, the interaction of gender x social support was statistically significant, *F*(1,136) = 5.99, *p* = 0.016, partial *η*^2^ = 0.042. The mean score for low social support in male caregivers was 91.83 (SD = 8.77) and that of the female caregivers was 82.09 (SD = 11.90). The mean score for high social support was 91.67 (SD = 10.73) for male caregivers and 91.52 (SD = 10.47) for female caregivers. Therefore, as Figure 1 shows, social support plays a moderating role between gender and resilience. Male caregivers presented similar resilience scores regardless of whether they were in the low or high social support groups, whereas in the case of female caregivers, they presented similar resilience score to male caregivers only if their social support was high.

### 3.4. Moderated Mediation

**Figure 2:**
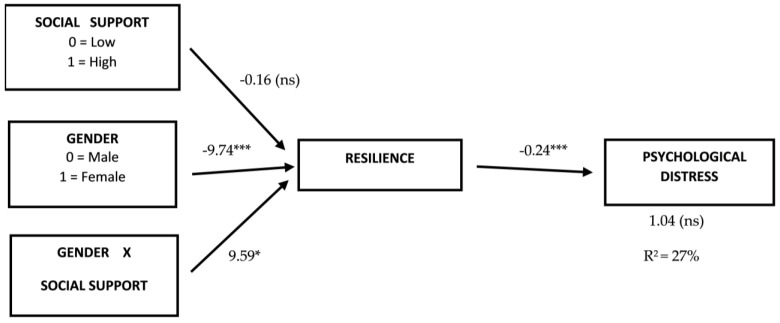
Mediation of resilience and moderation of social support on resilience between gender and psychological distress. Unstandardized β values. ns = (non-significant); R^2^ = Explained variance. * = *p* ≤ 0.05; *** = *p* ≤ 0.001.

Moderation and mediation analyses were performed based on ordinary least-squared regression and the bootstrap method (10,000 bootstrap samples). The indirect mediating/moderating effects of variables on the bootstrap method were evaluated based on whether the point estimate of the mediating/moderating variables was zero within a 95% bias-corrected and accelerated confidence interval. Consequently, a bootstrapping of 95% confidence not containing the zero-interval is considered statistically significant. The indirect effects of resilience between gender and psychological distress, resilience being moderated by social support, are presented in Figure 2. The mediation role of resilience between gender and psychological distress produces a full mediation due to the significant beta (*Beta* = 2.71; *p* < 0.01) that loses the significant effect when resilience was introduced into the equation (*Beta* = −1.04, *p* = 0.242). The model explains 27% of the variance.

Table 4 shows the conditional effect of gender on the low and high social support groups. We can see that only when social support is low (*Beta* = 2.31, 95% CI [1.06, 3.77]) does gender present with a significant effect. In other words, the moderating effect of social support between gender and psychological distress, with resilience as a mediator, is only significant when the level of social support is low. As can be seen in Figure 1, when social support is high, women and men showed similar resilience levels.

No mediation or moderation resulted in being significant in relation to the variable of burden.

## 4. Discussion

The results show that female caregivers present higher psychological distress and lower resilience than male caregivers; however, no significant differences have been found in terms of burden and social support. The percentages of burden are very high for both female and male caregivers of relatives with AD, with over 77% perceiving moderate and severe burden when caring for their relative, confirming previous studies [9,13].

Psychological distress had an important impact on more than half of the female caregivers in the study (56.2%), whereas only 38% of the male caregivers showed high scores. Several studies have presented similar results with high levels of psychological distress for the caregivers of family members with AD, especially women [7,9,10,11,12,13]. Caring for a family member with AD implies a heavy burden that affects women than men. Our results did not match other studies that found that female caregivers presented a higher burden than male caregivers [30]; however, it can be stated that both men and women showed a high level of burden, with no part of our sample presenting low burden, with over a half of the sample being in the mild to moderate level, and almost a quarter of it in the severe level of burden. Some studies found a greater impact on the mental health of caregivers when caring for relatives at more advanced stages of AD, especially in the variables of burden and psychological distress [43,44]; however, our results failed to confirm this finding. The high levels of burden presented by the AD caregivers in this study could explain the lack of significant differences in terms of social support, resilience, or psychological distress between caregivers caring for relatives at different stages of AD. Social support and resilience did not present gender differences and, consequently, we could not confirm other works where females perceived higher social support and resilience than male caregivers [32].

The most significant result of this study is the role played by social support and resilience in lowering the impact of mental health problems in relation to gender. Both variables had an important protecting role in terms of psychological distress, with social support showing a moderating role and resilience a mediating one. However, these variables had different roles depending on the gender of the caregiver. Female caregivers need higher social support to present similar resilience to male caregivers. The role of social support seems to be more limited in male caregivers. This result confirms that both social support and resilience are protective factors of psychological distress variables [24,25,26,27,28,29]. However, when the gender of the caregivers is considered, social support is a very important variable in the development of resilience in female caregivers, something that seems to be not so important in male caregivers.

Kim et al. [45] confirmed the different impacts of caregiving on men and women caring for cancer patients. In this case, male caregivers appraised the caregiving experience as boosting their self-esteem, and female caregivers appraised the experience as more stressful than male caregivers. Hernandez-Padilla et al. [32] found that perceived social support was a mediating variable between perceived health and burden but only in female caregivers of relatives with AD, confirming the different effects of social support on male and female caregivers.

Several approaches have been proposed to explain gender differences in caregiving. The results from our study support the gender role theory, where women are overrepresented as caregivers as a consequence of socialized gender roles [14,15], in many cases the female caregiver population being twice that of male caregivers, as is the case in this study. Our work also highlights the modulating role of social support and the mediating role of resilience between gender and psychological distress, confirming the relevance of variables whose role is to modulate or mediate gender differences in mental health, as proposed in stress-coping theory [9,15,16,17,18,19].

This work has three limitations: firstly, it is a cross-sectional study, so causal inferences cannot be made. Secondly, the information has been gathered using self-reports, a method that may have biases, such as issues concerning social desirability, and finally, this study did not use a random sample, so it may not be representative of all Spanish AD caregivers. However, our study compared the profiles of caregivers of family members with Alzheimer’s disease and found that in the last decade in Spain, [6], similarities in the higher percentages of women, the fact that most caregivers are daughters and sons and the mean age of caregivers being between 50 and 60 years old. These similarities support the generalizability of our results.

In future research, a thorough examination of female caregivers’ stressors and their appraisal is necessary to develop resilience programs that consider the complexity of their caregiving experience. Systematic reviews and meta-analyses on interventions with caregivers of family members with Alzheimer’s family reported effects in decreasing psychological distress in a wide range of caregivers, including psychoeducational, mutual support, psychosocial, cognitive behavioral, or multicomponent (combination of support, psychotherapy, and educational) interventions, both at the group and individual levels, but requiring a minimum of eight sessions [46,47]. In this context, our results suggest that women could obtain better benefits when a social component is part of the intervention strategy.

## 5. Conclusions

The present work highlights the importance of gender in the study of psychological distress in the caregivers of AD family members. It shows that burden levels are high for both female and male caregivers and underlines the importance of social support as a key variable in association with gender to understand the mediating role of resilience in psychological distress. Although there are actual programs working on resilience in the caregivers of family members with AD [48,49], it seems that the effects are small or very small. A gender perspective should be taken to increase efficacy and help those who suffer greater impacts in terms of caregiving: women.

## Figures and Tables

**Table 1:** Demographic variables.

		*N*	%
Gender	Women	96	68.4
	Men	44	31.4
Education	Primary	69	49.3
	Secondary	50	35.7
	University	21	15.0
Living with/without a partner	With a partner	118	84.3
	Without a partner	22	15.7
Relationship with the AD person	Daughter/son	95	67.9
	Spouse	24	17.1
	Daughter/son in law	13	9.3
	Grandchild	5	3.6
	Niece/nephew	3	2.1
Person with AD stage	Mild	42	30.0
	Moderate	68	48.6
	Severe	30	21.4

**Table 2:** Means, standard deviations, Student’s *t*, Cohen’s *d* (Male = 44; Female = 96).

Variables	Gender	Mean	SD	*t*	*d*
1. Psychological distress	Male	13.34	4.79		
	Female	15.46	5.75	−2.13 *	0.40
2. Burden	Male	50.25	10.53		
	Female	52.10	13.03	−0.83	---
3. Social support	Male	40.68	7.78		
	Female	41.54	9.15	−0.54	---
4. Resilience	Male	91.75	9.64		
	Female	87.50	12.05	2.20 *	0.42

* = *p* ≤ 0.05.

**Table 3:** Relationships between psychological distress, burden, social support and resilience. (Male caregivers on the below left and female on the above right part).

Variables	1	2	3	4
1. Psychological distress	---	0.52 ***	−0.42 ***	−0.55 ***
2. Burden	0.64 ***	---	−0.37 ***	−0.40 ***
3. Social support	−0.24	−0.42 **	---	0.42 ***
4. Resilience	−0.30 *	−0.26	0.02	---

* = *p* ≤ 0.05; ** = *p* ≤ 0.01; *** = *p* ≤ 0.005.

**Table 4:** Conditional indirect effect of gender on psychological distress at the values of social support (0 = low social support; 1 = high social support) with resilience as mediator.

MEDIATOR	Social Support	Effect	Boot SE	BootLLCI	BootULCI
Resilience	0	2.31	0.69	1.06	3.77
Resilience	1	0.04	0.65	−1.28	1.30
INDEX OF MODERATED MEDIATION					
Resilience		−2.27	0.92	−4.29	−0.66

## Data Availability

The datasets generated and analysed during the current study are available from the corresponding author upon reasonable request.

## References

[1] WHO (2017). Global Action Plan on the Public Health Response to Dementia 2017–2025.

[2] WHO (2023). Dementia.

[3] Garre J. (2018). Epidemiología de la enfermedad de Alzheimer y otras demencias [Epidemiology of Alzheimer’s disease and other dementias]. Rev. Neurol..

[4] Tahami Monfared A.A., Byrnes M.J., White L.A., Zhang Q. (2022). Alzheimer’s Disease: Epidemiology and Clinical Progression. Neurol. Ther..

[5] INE, Instituto Nacional de Estadística Encuesta de Discapacidad, Autonomía Personal y Situaciones de Dependencia (EDAD 2020) [Survey on Disability, Personal Autonomy and Dependency Situations]; Spanish Government: 2022. https://www.ine.es/dyngs/INEbase/es/operacion.htm?c=Estadistica_C&cid=1254736176782&menu=resultados&idp=1254735573175#!tabs-1254736195764.

[6] Civiriain-San Miguel L., Moré-Rubio B. (2021). Systematic review of the profile of Alzheimer’s family caregivers over the last ten years. PortalesMedicos.

[7] Sallim A.S., Sayampanathan A.A., Cuttilan A., Ho R.C.M. (2015). Prevalence of mental health disorders among caregivers of patients with Alzheimer disease. JAMDA.

[8] Swinkels J.C., Broese M.I., de Boer A., Tilburg T.G.V. (2019). Male and female partner-caregivers burden: Does it get worse over time?. Gerontologist.

[9] Akpinar B., Küçükgüçlü Ö., Yener G. (2011). Effects of gender on burden among caregivers of Alzheimer’s patients. J. Nurs. Scholarsh..

[10] Mathias K., Kermode M., San Sebastian M., Davar B., Goicolea I. (2019). An asymmetric burden experiences of men women as caregivers of people with psychosocial disabilities in rural North India. Transcult. Psychiatry.

[11] Pöysti M.M., Laakkonen M.L., Strandberg T., Savikko M., Tilvis R.S., Eloniemi-Sulkava U., Pitkälä K.H. (2012). Gender differences in dementia spousal caregiving. Int. J. Alzheimers Dis..

[12] Scott C.B. (2013). Alzheimer’s disease caregiver burden: Does resilience matter?. J. Hum. Behav. Soc. Environ..

[13] Takano M., Arai H. (2005). Gender differences and caregivers’ burden in early-onset Alzheimer disease. Psychogeriatrics.

[14] Papastavrou E., Tsangari H., Kalokerinou A., Papacostas S.S., Sourtzi P. (2009). Gender issues in caring for demented relatives. Health Sci. J..

[15] Pinquart M., Sörensen S. (2006). Gender differences in caregiver stressors, social resources, and health: An updated meta-analysis. J. Gerontol. B Psychol. Sci. Soc. Sci..

[16] del-Pino-Casado R., Frías-Osuna A., Palomino-Moral P.A., Martínez-Riera R.J. (2012). Gender differences regarding informal caregivers of older people. J. Nurs. Scholarsh..

[17] Yee J.L., Schulz R. (2000). Gender differences in psychiatric morbidity among family caregivers: A review and analysis. Gerontologist.

[18] Kim H., Chang M., Rose K., Kim S. (2012). Predictors of caregiver burden in caregivers of individuals with dementia. J. Adv. Nurs..

[19] Wallsten S.S. (2000). Effects of caregiving, gender, and race on the health, mutuality, and social support of older couples. J. Aging Health.

[20] Campbell P., Wright J., Oyebode J., Job D., Crome P., Bentham P., Jones L., Lendon C. (2008). Determinants of burden in those who care for someone with dementia. Int. J. Geriatr. Psychiatry.

[21] Sharma N., Chakrabarti S., Grover S. (2016). Gender differences in caregiving among family-caregivers of people with mental illnesses. World J. Psychiatry.

[22] Gallicchio L., Siddiqi N., Langenberg P., Baumgarten M. (2002). Gender differences in burden and depression among informal caregivers of demented elders in the community. Int. J. Geriatr. Psychiatry.

[23] Mohamed S., Rosenheck R., Lyketsos C.G., Schneider L.S. (2010). Caregiver burden in Alzheimer disease: Cross-sectional and longitudinal patient correlates. Am. J. Geriatr. Psychiatry.

[24] Chen X., Mao Y., Kong L., Li G., Xin M., Lou F., Li P. (2016). Resilience moderates the association between stigma and psychological distress among family caregivers of patients with schizophrenia. Personal. Individ. Differ..

[25] Durán-Gómez N., Guerrero-Martín J., Pérez-Civantos D., López-Jurado C.F., Palomo-López P., Cáceres M.C. (2020). Understanding resilience factors among caregivers of people with Alzheimer’s disease in Spain. Psychol. Res. Behav. Manag..

[26] Fang L., Dong M., Fang W., Zheng J. (2022). Relations between care burden, resilience, and depressive symptoms among the main family caregivers of stroke patients: A cross-sectional study. Front. Psychiatry.

[27] George E.S., Kecmanovic M., Meade T., Kolt G.S. (2020). Psychological distress among carers and moderating effects of social support. BMC Psychiatry.

[28] Mulud Z.A., McCarthy G. (2017). Caregiver burden among caregivers of individuals with severe mental illness: Testing the moderation and mediation models of resilience. Arch. Psychiatr. Nurs..

[29] Wilks S.E., Croom B. (2008). Perceived stress and resilience in Alzheimer’s disease caregivers: Testing moderation and mediation models of social support. Aging Ment. Health.

[30] Díaz M., Estévez A., Momeñe J., Ozerinjauregi N. (2019). Social support in the relationship between perceived informal caregiver burden and general health of female caregivers. Ansiedad y Estrés.

[31] Ong H.L., Vaingankar J.A., Abdin E., Sambasivam R., Fauziana R., Tan M., Chong S.A., Goveas R.R., Chiam P.C., Subramaniam M. (2018). Resilience and burden in caregivers of older adults: Moderating and mediating effects of perceived social support. BMC Psychiatry.

[32] Hernández-Padilla J.M., Ruiz-Fernández M.D., Granero-Moina J., Ortíz-Amo R., López-Rodríguez M.M., Fernández-Solá C. (2021). Perceived health, caregiver overloads and perceived social support in family caregiver of patients with Alzheimer’s: Gender differences. Health Soc. Care Community.

[33] Dias R., Santos R.L., Barroso de Sousa M.F., Lima Nogueira M.M., Torres B., Belfort T., Dourado M.C.M. (2015). Resilience of caregivers of people with dementia: A systematic review of biological and psychological determinants. Trends Psychiatry Psychother..

[34] Kimura N.R.S., Simões-Neto J.P., Santos R.L., Tourinho-Baptista M.A., Portugal G., Johannessen A., Barca M.L., Engedal K., Laks K., Rodrigues V.M. (2019). Resilience in carers of people with young-onset Alzheimer disease. J. Geriatr. Psychiatry Neurol..

[35] Palacio C., Krikorian A., Gómez-Romero M.J., Limonero J.T. (2020). Resilience in Caregivers: A Systematic Review. Am. J. Hosp. Palliat. Care..

[36] Goldberg D.P., Gater R., Sartorius N., Ustun T.B., Piccinelli M., Gureje O. (1997). The validity of two versions of the GHQ in the WHO study of mental illness in general health care. Psychol. Med..

[37] Lundin A., Åhs J., Åsbring N., Kosidou K., Dal H., Tinghög P., Saboonchi F., Dalman C. (2017). Discriminant validity of the 12-item version of the general health questionnaire in a Swedish case-control study. Nord. J. Psychiatry.

[38] Zarit S.H., Zarit J.M. (1987). The Memory and Behavior Problems Checklist and the Burden Interview (Technical Report).

[39] Broadhead W.E., Gehlbach S.H., de Gruy F.V., Kaplan B.H. (1988). The Duke-UNC Functional Social Support Questionnaire: Measurement of social support in family medicine patients. Med. Care.

[40] Connor K.M., Davidson J.R.T. (2003). Development of a new resilience scale: The Connor-Davidson Resilience Scale (CD-RISC). Depress. Anxiety.

[41] Hayes A.F. (2018). Introduction to Mediation, Moderation, and Conditional Process Analysis: A Regression-Based Approach.

[42] Feldman H.H., Woodward M. (2005). The staging and assessment of moderate to severe Alzheimer disease. Neurology.

[43] Koca E., Taşkapilioğlu Ö., Bakar M. (2017). Caregiver burden in different stages of Alzheimer’s disease. Arch. Neuropsychiatry.

[44] Mukherjee A., Biswas A., Roy A., Biswas S., Gangopadhyay G., Das S.K. (2018). Behavioural and psychological symptoms of dementia: Correlates and impact on caregiver distress. Dement. Geriatr. Cogn. Disord. Extra.

[45] Kim Y., Baker F., Spillers R.L. (2007). Cancer caregivers’quality of life: Effects of gender, relationship, and appraisal. J. Pain Symptom Manag..

[46] Hansen N.H., Bjerrekær L., Pallesen K.J., Juul L., Fjorback L.O. (2022). The effect of mental health interventions on psychological distress for informal caregivers of people with mental illness: A systematic review and meta-analysis. Front. Psychiatry.

[47] Cheng S.T., Zhang F.A. (2020). Comprehensive meta-review of systematic reviews and meta-analyses on nonpharmacological interventions for informal dementia caregivers. BMC Geriatr..

[48] Cheng S.T., Li K.K., Or P.P.L., Losada A. (2022). Do caregiver interventions improve outcomes in relatives with dementia and mild cognitive impairment?. A comprehensive systematic review and meta-analysis. Psychol. Aging.

[49] Ghaffari F., Rostami M., Fotokian Z., Hajiahmadi M. (2019). Effectiveness of resilience education in the mental health of family caregivers of elderly patients with Alzheimer disease. Iran. J. Psychiatry Behav. Sci..

